# Betulin Phosphonates; Synthesis, Structure, and Cytotoxic Activity

**DOI:** 10.3390/molecules21091123

**Published:** 2016-08-26

**Authors:** Elwira Chrobak, Ewa Bębenek, Monika Kadela-Tomanek, Małgorzata Latocha, Christian Jelsch, Emmanuel Wenger, Stanisław Boryczka

**Affiliations:** 1 Department of Organic Chemistry, School of Pharmacy with the Division of Laboratory Medicine in Sosnowiec, Medical University of Silesia in Katowice, 4 Jagiellońska Str., 41-200 Sosnowiec, Poland; ebebenek@sum.edu.pl (E.B.); mkadela@sum.edu.pl (M.K.-T.); boryczka@sum.edu.pl (S.B.); 2 Department of Cell Biology, School of Pharmacy with the Division of Laboratory Medicine in Sosnowiec, Medical University of Silesia in Katowice, 8 Jedności Str., 41-200 Sosnowiec, Poland; mlatocha@sum.edu.pl; 3 Laboratoire Cristallographie Résonance Magnétique et Modélisations, Faculté des Sciences et Technologies, CNRS, Université de Lorraine, BP 70239, 54506 Vandoeuvre-les-Nancy, France; christian.jelsch@univ-lorraine.fr (C.J.); emmanuel.wenger@univ-lorraine.fr (E.W.)

**Keywords:** betulin, phosphonate, *Z* isomer, *E* isomer, crystallographic analysis, antiproliferative activity

## Abstract

Betulin derivatives are a widely studied group of compounds of natural origin due to their wide spectrum of biological activities. This paper describes new betulin derivatives, containing a phosphonate group. The allyl-vinyl isomerization and synthesis of acetylenic derivatives have been reported. Structural identification of products as *E* and *Z* isomers has been carried out using ^1^H-, ^13^C-, ^31^P-NMR, and crystallographic analysis. The crystal structure in the orthorhombic space group and analysis of crystal packing contacts for 29-diethoxyphosphoryl-28-cyclopropylpropynoyloxy-lup-20*E*(29)-en-3β-ol **8a** are reported. All new compounds were tested in vitro for their antiproliferative activity against human T47D (breast cancer), SNB-19 (glioblastoma), and C32 (melanoma) cell lines.

## 1. Introduction

**Scheme 1:**
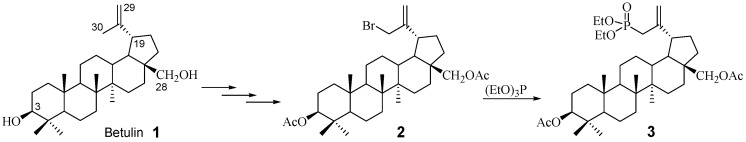
Synthesis of 3β,28-diacetoxy-30-diethoxyphosphoryl-lup-20(29)-ene **3**.

Natural plant products are an important source of the exploration and development of new anticancer drugs. Betulin (lup-20(29)-ene-3β,28-diol) **1** (Scheme 1), known for over 200 years, pentacyclic triterpene is isolated from a bark of many species of birch. The high content of betulin 1 in white birch bark (up to 30%) and the presence of two hydroxyl groups at C3 and C28 and an isopropenyl group at C19 results in compound **1** being a good material for the preparation of new derivatives with a broad spectrum of biological activities, such as anticancer, antiviral, antimalarial, antibacterial, anti-inflammatory, and hepatoprotective [1]. It was reported that the introduction of substituents containing a triple bond into the betulin **1** results in derivatives with increased cytotoxicity, and provides a starting point for further modification of the compound [2,3,4].

As an extension of our study on the development of novel betulin derivatives with potential anticancer activity, we became interested in the synthesis and evaluation of cytotoxicity of betulins containing a phosphonate group at the C30 position. Phosphonates are widely distributed compounds in living organisms, performing a variety of functions. Introduction of phosphonate, as bioisosteric group to phosphate, has been used in the development of drugs or searching for new therapeutic substances regarding their stability in the conditions of the enzymatic hydrolysis [5,6]. Moreover, among phosphonates, one can find active substances such as antiviral, antibacterial, antitumor, and drugs used in the treatment of osteoporosis [7,8,9,10]. Penetration of biological membranes of the free phosphonic acids is not as effective as for phosphonic esters due to the high negative charge of the phosphoryl moiety [11]. This is the reason why phosphonic acid ester synthesis has an important meaning in the designing of new phosphonates. 

In this paper, we have described the synthesis and structure of new derivatives of betulin containing phosphonate groups. Furthermore, we performed the synthesis of acetylenic derivatives of betulin phosphonate. The structures of the obtained compounds were confirmed based on the ^1^H-, ^13^C-, and ^31^P-NMR spectra. An X-ray method was used to determine the structure of one of the isomers formed in the hydrolysis reaction. To our knowledge the synthesis and crystal structure of phosphonate betulin have not been reported until now. The newly-obtained compounds were investigated for their cytotoxic activity towards human cell lines such as glioblastoma (SNB-19), breast tumour (T47D) and melanoma (C32).

## 2. Results and Discussion

### 2.1. Chemistry

In this work we have synthesized new betulin derivatives with a phosphonate group in substituent at the C19. As a starting substrate, we used the 3β,28-diacetoxy-30-bromo-lup-20(29)-ene **2** obtained from betulin **1** according to the method described by Sun et al. [12]. The synthesis of the betulin phosphonate **3** was carried out in good yield (76%) via the Michaelis-Arbuzov reaction of compound **2** with triethyl phosphite (Scheme 1).

**Scheme 2:**
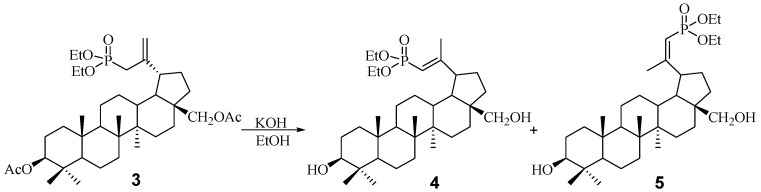
Hydrolysis of compound **3**.

In order to perform any further modifications of the compound **3**, a deprotection of the hydroxyl groups in the positions C3 and C28 was necessary. In the literature, several methods of performing this reaction are described for a variety of betulin derivatives. Generally, this process is carried out under mild basic conditions (room temperature, sodium hydroxide, methanol/tetrahydrofuran/water), and a relatively long reaction time (19–24 h) [12,13]. Increasing the temperature of the reaction makes it possible to shorten the time. Hydrolysis of 3β,28-diacetoxy-30-diethoxyphosphoryl-lup-20(29)-ene **3** carried out in the presence of potassium hydroxide and refluxing ethanol for two hours unexpectedly gave a mixture of two isomers, **4** and **5**, (Scheme 2) in a 1:0.2 ratio (based on ^31^P-NMR) which were separated by column chromatography on silica gel (dichloromethane/ethanol, 15/1, *v*/*v*) giving the pure compounds **4** and **5** in yields of 64% and 13%, respectively.

The signal of phosphorus atom in the ^31^P-NMR spectrum of the substrate **3** is located at 27.73 ppm, while the chemical shifts of the products are 18.54 ppm and 17.92 ppm for **4** and **5**, respectively. Such a pronounced upfield shift of the ^31^P signal can be caused by the unsaturation at the site of the alpha carbon [14].

The structures of compounds **4** and **5** were confirmed by their spectral data (^1^H- and ^13^C-NMR). Comparing the ^1^H-NMR spectra of both substrate (**3**) and products (**4**, **5**), it is noticed that the signals from the two protons H29, presenting as two singlets (5.07 and 5.01 ppm) in the spectrum of **3**, there is a doublet signal derived from one proton at 5.42 ppm (^2^*J*_HP_ = 18.6 Hz) and at 5.28 ppm (^2^*J*_HP_ = 19.2 Hz) for **4** and **5**, respectively (Figure S1). Similarly, in the ^13^C-NMR spectra, the signal of the carbon atom connected with the phosphoryl group (C29) for compound **4** is present at 111.0 ppm in the form of a doublet with a coupling constant ^1^*J*_CP_ = 193 Hz, whereas in the spectrum of compound **5**, a doublet is located at 111.9 with a coupling constant ^1^*J*_CP_ = 189 Hz. The ^1^H- and ^13^C-NMR coupling constants were consistent with the values reported for the other vinyl phosphonates [15].

**Scheme 3:**
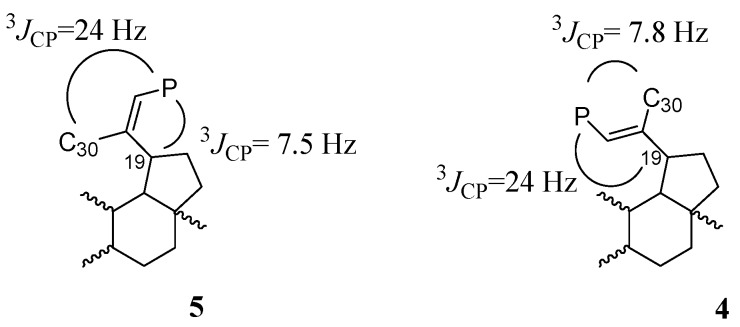
Determination of the stereochemistry for isomers **4** and **5** on the basis of coupling constants (^3^*J*_CP_).

The above results suggested that the performed reaction of hydrolysis of the acetyl groups additionally led to the isomerization in the isopropenyl moiety. Several literature data have shown that then allyl phosphonate diesters undergo isomerization to the vinyl phosphonate diesters. This process occurs in various palladium catalysed nucleophilic substitution reactions, thermal rearrangements and under basic conditions [16,17,18,19,20,21]. The stereochemical assignments of the new obtained vinylic derivatives were also determined by the carbon phosphorus coupling constants (^3^*J*_CP_). For the olefin phosphonates, this constant is typical and dependent on the spatial structure. It has been reported that for various vinylphosphonates, the *cis* ^3^*J*_CP_ coupling constant is generally smaller than *trans* ^3^*J*_CP_ in the ^13^C-NMR and they are equal to 6–8 Hz and 20–25 Hz, respectively [22,23]. For isomer **5**, signals of the C30 and C19 carbon atoms in the ^13^C-NMR spectrum are located at δ_C_: 21.5 and 43.8 ppm, respectively (Figure S2). The small ^3^*J*_CP_ (7.5 Hz) and the large ^3^*J*_CP_ (24 Hz) coupling constant are consistent with the *cis* and *trans* configurations of C19 or C30 in relation to the phosphorus atom, as shown in Scheme 3. Olefin stereochemistry of the compound **5** was determined as (*Z*)-isomer. For compound **4**, signals of C30 and C19 are observed at δ_C_: 17.5 (d, ^3^*J*_CP_ = 7.8 Hz) and 51.5 (d, ^3^*J*_CP_ = 24 Hz), respectively.

**Scheme 4:**
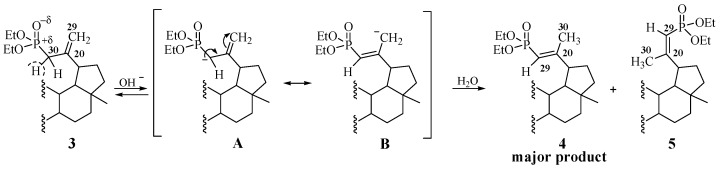
Mechanism of allyl-vinyl phosphonate isomerization under basic conditions.

On the basis of spectroscopic analysis and taking into account the conditions of the carried out hydrolysis reaction, we have proposed the following mechanism for the observed allyl-vinyl isomerization, shown in Scheme 4.

The electron withdrawing effect of the phosphonate moiety increases the acidity of *α* protons. Under the basic conditions, a reaction of deprotonation occurs which leads to the formation of the allylic carbanion. The obtained carbanion (**A** and **B** mesomeric structures) is stabilized by resonance with π electrons of multiple bond [24,25,26]. Is seems that, the vinyl phosphonate **4**, as the final product of the reaction, is thermodynamically more stable than the structure with the double bond in the terminal position (**3** in Scheme 4). The allyl-vinyl phosphonate isomerization under the basic conditions also generates a small amount of (*Z*)-isomer (compound **5**).

The stereochemistry of compound **4** was indirectly determined by an analysis of the crystal structure of its acetylenic derivative **8a**.

**Scheme 5:**
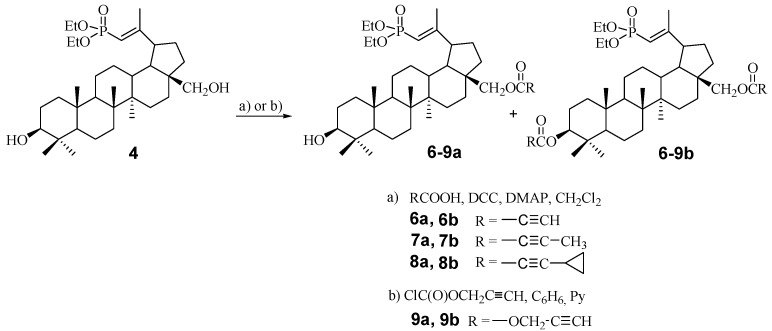
Synthesis of acetylenic derivatives of 29-diethoxyphosphoryl betulin **4**.

The treatment of **4** with acetylenic acid (propiolic, 2-butynoic, 3-cyclopropyl-2-propiolic) in dichloromethane in the presence of *N*,*N’*-dicyclohexylcarbodiimide (DCC) and dimethylaminopyridine (DMAP) gave corresponding 28-alkynyl ester of 29-diethoxyphosphoryl betulin **6a**–**8a** in 74%–87% yield, according to the method described previously (Scheme 5) [2]. The second product in this reaction was a small amount of 3,28-diester of betulin **6b**–**8b** (2%–13% yield).

Additionally, the reaction of 29-diethoxyphosphoryl-lup-20*E*(29)-en-3β,28-diol **4** with propargyl chloroformate, in benzene in the presence of pyridine [2], to afford the alkynyloxycarbonyl derivatives **9a** and **9b** was performed.

Isomer **5** is formed in the reaction in small quantities; therefore, it was not considered to use for further transformations.

### 2.2. Crystal Structure of 29-Diethoxyphosphoryl-28-cyclopropylpropynoyloxy-lup-20E(29)-en-3*β*-ol ***8a***

**Figure 1:**
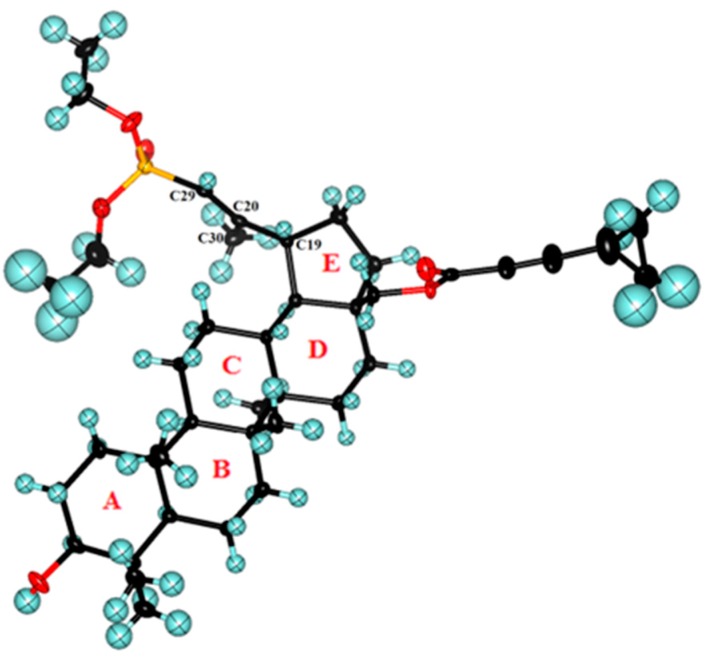
View of the molecular structure of compound **8a** (*E*-isomer). The thermal ellipsoids are shown at 50% probability level.

The six-membered rings (A, B, C, D) (for ring numbering see Figure 1) in the pentacyclic system of 29-diethoxyphosphoryl-28-cyclopropylpropynoyloxy-lup-20*E*(29)-en-3β-ol **8a**, adopt a chair conformation and all of the ring junctions are trans-fused. Such ring conformation is similar to that found in solvates of betulin [27], betulinic acid [28], and 28-*O*-propynoylbetulin [2].

The cyclopentane E-ring adopts an envelope conformation with the C17 atom being displaced from the C18-C19-C21-C22 plane about 0.694 Å. The methyl groups with C24, C25, C26, and C27 atoms are in axial positions, whereas that with C23 is equatorial. The substituent attached to the atom C17 is in axial orientation, while the hydroxyl group at the C3 and the substituted isopropenyl group at C19 occupy the equatorial position. The hydrocarbon side chains bound on the ester and phosphonate moieties display higher thermal motion than the triterpene core of the molecule (Figure 1). The orientation of the isopropenyl group, relative to the five-membered ring, can be described by the torsion angle C29-C20-C19-C21, which is equal to −114.8(2)°. The substituents attached to sp^2^ hybridized carbons C20 and C29 are in trans configuration and torsion angles P-C29-C20-C19 and P-C29-C20-C30 are equal to −176.7(2)° and 3.4(4)°, respectively.

This information confirmed that the compound **8a** possessed the (*E*)-configuration; therefore, it was concluded that the compound **4**, which was the substrate for the synthesis of the derivative **8a**, also has the (*E*)-configuration.

The crystal contacts have been analyzed with the Hirshfeld surface methodology [29]. The majority of the contacts in the crystal are of Hc…Hc type (Table 1) as most of the molecular surface is constituted by aliphatic hydrocarbons (Hc). The molecule has three strong hydrogen-bond acceptors (oxygen atoms of type O=P, O=C and hydroxyl) and one strong donor (hydroxyl group). A driving force in the stabilization of this crystal packing is the formation of the single strong P=O···H-O hydrogen bond between the phosphonate and the hydroxyl group. In addition, there are seven weak C-H···O hydrogen bonds with five of the six oxygen atoms present in the structure (Table S2). After Hc···Hc, the most abundant contacts in the crystal packing are Hc···C and Hc···O interactions (Table 1). Self-contacts of O···O and Ho···Ho types are totally avoided in the packing as these interactions between charged atoms are electrostatic unfavorable. The enrichment ratios of contacts between pairs of chemical species are shown in Table 1; the ratios > 1 or < 1 indicate if contacts in the crystal packing are favored or disfavored, respectively [30]. Carbon is exposed on the molecular surface essentially in the C≡C-C (ester) region and C…Hc is the only contact type involving carbon which is statistically favored (enrichment of 1.1 in Table 1) in this crystal structure. The strong enrichment of the O···Ho contact highlights its importance in the crystal. The two most abundant interactions, the hydrophobic Hc···Hc and the weakly polar O···Hc contacts are neither favored, neither disfavored, with enrichment values close to unity. All other contact types (except the marginal P···Hc contacts) are impoverished.

### 2.3. Cytotoxic Activity

The newly-synthesized compounds **3**–**5**, **6a**–**9a**, and **6b**–**9b** were tested for their in vitro antiproliferative activity against human cancer lines T47D (breast cancer), SNB-19 (glioblastoma), and C32 (melanoma) using the WST-1 assay. The obtained results were expressed as the concentration of compound (µg/mL), which inhibits the proliferation of 50% of tumor cells, as compared to the control untreated cells (IC_50_). Betulin **1** (the parent material) and cisplatin (the cytotoxic agent) were used as reference compounds. The results of these studies are summarized in Table 2.

3,28-Diacetyl betulin derivative **3** containing the phosphonate group at C30 position exhibited low activity against the human breast cancer (T47D) and glioblastoma (SNB-19), while it efficiently inhibited proliferation of melanoma cell lines (C32). As showed in Table 2, for all tested lines, the introduction of the phosphonate group in C29 position (compounds **4** and **5**) resulted in an increase of activity in comparison with betulin **1**.

Cytotoxicity of *E* and *Z* isomers of 29-phosphonate betulin (compounds **4** and **5**, respectively) were comparable in the case of the C32 cancer line. In relation to the human breast cancer (T47D), *Z*-isomer had nearly seven times higher cytotoxicity than the *E*-isomer.

Among the acetylenic derivatives of the betulin phosphonate, the highest activity showed 29-diethoxyphosphoryl-28-propynoyloxy-lup-20*E*(29)-en-3β-ol **6a** which contains the small alkynyl group with a terminal C≡C bond. As can be seen, compound **6a** also exhibited a higher cytotoxic activity in a comparison with the reference compound cisplatin for all tested cell lines. The ester derivatives **6a**–**8a** and **6b**–**8b** were the most active towards melanoma cell lines (C32) with IC_50_ values in the range 0.38–9.44 µg/mL. In the series of monosubstituted derivatives **6a**–**8a** the rank order of antiproliferative activity against C32 and T47D cell lines is the following: **6a** > **8a** > **7a**.

The replacement of the ester group by a formate moiety resulted in a significant decrease in activity of the formed compounds (**9a**,**b**) against human breast cancer (T47D). The propargyloxycarbonyl derivative **9b** exhibited low activity towards C32 while, relative to the SNB-19 line, revealed higher activity than the reference compound cisplatin.

## 3. Materials and Methods

### 3.1. General Techniques

Melting points of compounds were obtained in open capillary tubes on a Boetius melting point apparatus without correction.

^1^H-NMR, ^13^C-NMR, and ^31^P-NMR spectra were measured on a Bruker AVANCE III HD 600 spectrometer (Bruker, Billerica, MA, USA), as deuterated solvent was used (CDCl_3_). The spectra for ^1^H, ^13^C, and ^31^P atoms were recorded at 600 MHz, 150 MHz, and 243 MHz, respectively. Chemical shift (δ) values are reported in parts per million (ppm) relative to the external standards and the coupling constants (*J*) are presented in Hertz. Multiplicity is designated as singlet (s), doublet (d), and multiplet (m). Infrared spectra (KBr, pellet) were recorded in the range of 4000–1000 cm^−1^ at 295K using the IRAffinity-1 FTIR spectrometer (Shimadzu Corporation, Kyoto, Japan).

HR mass spectra were recorded with Bruker Impact II (Bruker, Billerica, MA, USA). Calculation of the theoretical molecular mass for compounds was performed using “The Exact Mass Calculator, Single Isotope Version” (http://www.sisweb.com/referenc/tools/exactmass.htm (Ringoes, NJ, USA).

The progress of reaction was monitored by TLC using silica gel 60 254F plates (Merck, Darmstadt, Germany) and developed in a mixture of chloroform and ethyl acetate (7:3, *v*/*v*) or dichloromethane and ethanol (15:1, *v*/*v*). The spots were visualized by spraying with a solution of 5% sulfuric acid and heating to 100 °C. Purification of the compounds was carried out by column chromatography (silica gel 60, <63 μm, Merck) in the solvent system indicated. All reactions solvents were dried and purified according to usual procedures.

3β,28-Diacetoxy-30-bromo-lup-20(29)-ene **2** was obtained according to described method. ^1^H- and ^13^C-NMR spectra data for compound **2** were consistent with the literature information [12].

### 3.2. Procedure for the Synthesis of 3β,28-Diacetoxy-30-diethoxyphosphoryl-lup-20(29)-ene ***3***

To a round bottom flask equipped with a reflux condenser, protected from moisture and under argon atmosphere 3β,28-diacetoxy-30-bromo-lup-20(29)-ene **2** (0.61 g, 1 mmol) and triethyl phosphite (3.4 mL, 20 mmol) were added. The reaction was carried out at reflux for 6 h. Then the excess of triethyl phosphite was distilled off and the residue was concentrated to dryness on a rotary evaporator. The resulting product was washed with hexane and then purified by column chromatography (SiO_2_, chloroform/acetone, 7: 3 *v*/*v*).

*3*β*,28-Diacetoxy-30-diethoxyphosphoryl-lup-20(29)-ene* (**3**). Yield: 76%, m.p. 152–155 °C, R_f_ 0.38 (SiO_2,_ chloroform/ethyl acetate, 7/3, *v*/*v*). ^1^H-NMR (CDCl_3_; 600 MHz) δ_H_: 0.80 (1H, m, H5), 0.85 (3H, s, CH_3_), 0.86 (6H, s, 2× CH_3_), 0.99 (3H, s, CH_3_), 1.05 (3H, s, CH_3_), 1.34 (6H, m, 2× OCH_2_C*H*_3_), 0.81–2.10 (24H, m, CH, CH_2_), 2.06 (3H, s, COC*H*_3_), 2.09 (3H, s, COC*H*_3_), 2.46 (1H, m, H19), 2.60 (2H, m, H30), 3.83 (1H, d, *J* = 10.8 Hz, H28), 4.13 (4H, m, 2× OC*H*_2_CH_3_), 4.28 (1H, d, *J* = 10.8 Hz, H28), 4.49 (1H, m, H3), 5.01 (1H, br. s, H29), 5.07 (1H, br. s, H29). ^13^C-NMR (CDCl_3_;150 MHz); δ_C_: 14.1; 14.8; 16.0; 16.1; 16.4; 16.5; 18.2; 20.9; 21.0; 21.3; 23.7; 26.4; 27.0; 27.9; 29.8; 31.6; 34.1; 34.2; 37.0; 37.4; 37.8; 38.4; 40.9; 42.7; 46.3; 49.9; 50.2; 55.4; 61.8; 61.9; 62.7; 80.9; 112.8; 144.6; 171.0; 171.5. ^31^P-NMR (CDCl_3_; 243 MHz) δ_P_: 27.73. IR (KBr, cm^−1^) ν_max_: 1741(C=O), 1248 (P=O), 961 (P-O-R), 784 (P-C). HR-MS (APCI, negative), *m/z* calcd. for C_38_H_63_O_7_P [(M − H)^−^] 661.4233, found 661.4228.

### 3.3. Procedure for the Hydrolysis of 3β,28-Diacetoxy-30-diethoxyphosphoryl-lup-20(29)-ene ***3***

Compound **3** (0.66 g, 1 mmol) was refluxed with KOH (0.31 g, 5.5 mmol) in ethanol (10 mL) for 2 h. After cooling to room temperature, the reaction mixture was concentrated to dryness under reduced pressure, the residue was triturated with water and filtered. The crude product was separated by column chromatography (SiO_2_, dichloromethane/ethanol, 15:1, *v*/*v*) to give the pure products **4** and **5**.

*29-Diethoxyphosphoryl-lup-20E(29)-en-3β,28-diol* (**4**). Yield: 64%, m.p. 120–123 °C, R_f_ 0.31 (SiO_2_, dichloromethane/ethanol, 15:1, *v*/*v*). ^1^H-NMR (CDCl_3_; 600 MHz) δ_H_: 0.70 (1H, m, H5), 0.78 (3H, s, CH_3_), 0.84 (3H, s, CH_3_), 0.99 (3H, s, CH_3_), 0.994 (3H, s, CH_3_), 1.07 (3H, s, CH_3_), 1.33 (6H, m, 2× OCH_2_C*H*_3_), 0.88–2.08 (25H, m, CH, CH_2_), 2.07 (3H, d, *J* = 3 Hz, H30), 2.45 (1H, m, H19), 3.21 (1H, m, H3), 3.32 (1H, d, *J* = 10.8 Hz, H28), 3.81 (1H, d, *J* = 10.8 Hz, H28), 4.05 (4H, m, 2× OC*H*_2_CH_3_), 5.42 (1H, d, ^2^*J*_HP_ = 18.6 Hz, H29). ^13^C-NMR (CDCl_3_; 150 MHz); δ_C_: 14.7; 15.4; 15.9; 16.1 (d, ^3^*J*_CP_ = 4.9 Hz, CH_3_) ; 16.4 (d, ^3^*J*_CP_ = 4.9 Hz, CH_3_); 16.9; 17.5 (d, ^3^*J*_CP_ = 7.8 Hz, C-30); 18.3; 20.8; 25.8; 26.9; 27.3; 28.0; 29.2; 29.9; 34.1; 34.2; 37.1; 38.6; 38.9; 40.9; 42.7; 47.9; 49.7; 50.3; 51.5 (d, ^3^*J*_CP_ = 24 Hz, C-19); 55.3; 60.4; 61.1; 78.9; 111.0 (d, ^1^*J*_CP_ = 193 Hz, C-29); 167.6. ^31^P-NMR (CDCl_3_; 243 MHz) δ_P_: 18.54. IR (KBr, cm^−1^) ν_max_: 3416 (OH), 1230 (P=O), 962 (P-O-R), 750 (P-C). HR-MS (APCI, negative), *m/z* calcd. for C_34_H_58_O_5_P [(M − H)^−^] 577.4022, found 577.4011.

*29-Diethoxyphosphoryl-lup-20Z(29)-en-3*β*,28-diol* (**5**). Yield: 13%, m.p. 126–128 °C, R_f_ 0.28 (SiO_2,_ dichloromethane/ethanol, 15:1, *v*/*v*). ^1^H-NMR (CDCl_3_; 600 MHz) δ_H_: 0.70 (1H, m, H5), 0.78 (3H, s, CH_3_), 0.84 (3H, s, CH_3_), 0.987 (3H, s, CH_3_), 0.99 (3H, s, CH_3_), 1.07 (3H, s, CH_3_), 1.33 (6H, m, 2× OCH_2_C*H*_3_), 0.85–2.10 (25H, m, CH, CH_2_), 1.87 (3H, br.s, H30), 3.50 (1H, m, H19), 3.20 (1H, m, H3), 3.43 (1H, d, *J* = 10.8 Hz, H28), 3.81 (1H, d, *J* = 10.8 Hz, H28), 4.06 (4H, m, 2× OC*H*_2_CH_3_), 5.28 (1H, dd, ^2^*J*_HP_ = 19.2 Hz, ^2^*J*_HH_ = 3.0 Hz, H29). ^13^C-NMR (CDCl_3_; 150 MHz) δ_C_: 14.7; 15.4; 15.9; 16.1; 16.4; 18.3; 20.8; 21.5 (d, ^3^*J*_CP_ = 24 Hz, C-30); 24.9; 27.0; 27.4; 28.0; 29.2; 29.3; 34.2; 34.3; 37.1; 37.2; 38.7; 38.9; 40.9; 42.7; 43.8 (d, ^3^*J*_CP_ = 7.5 Hz, C-19); 47.9; 49.8; 50.5; 55.3; 60.5; 61.1; 61.2; 78.9; 111.9 (d, ^1^*J*_CP_ = 189 Hz, C-29); 167.5. ^31^P-NMR (CDCl_3_; 243 MHz) δ_P_: 17.92. IR (KBr, cm^−1^) ν_max_: 3416 (OH), 1227 (P=O), 963 (P-O-R), 747 (P-C). HR-MS (APCI, negative), *m/z* calcd. for C_34_H_58_O_5_P [(M − H)^−^] 577.4022, found 577.4011.

### 3.4. General Procedure for the Synthesis of Acetylenic Derivatives of Betulin ***6a***–***8a*** and ***6b***–***8b***

A solution of compound **4** (0.29 g, 0.5 mmol) and 0.58 mmol of appropriate acid in 2.5 mL of dichloromethane was cooled down to −10 °C, and then, a solution of DCC (0.12 g, 0.59 mmol) and DMAP (0.005 g, 0.04 mmol) in 0.5 mL of dichloromethane was added dropwise. The mixture was stirred in an atmosphere of argon for 5 h in the cooling bath, and then at room temperature. After 24 h, the reaction mixture was filtered off and the filtrate was concentrated to dryness on a rotary evaporator. The products were separated by column chromatography (SiO_2_, dichloromethane/ethanol, 15: 1, *v*/*v*).

*29-Diethoxyphosphoryl-28-propynoyloxy-lup-20E(29)-en-3*β*-ol* (**6a**). Yield: 74%, m.p. 190–191 °C, R_f_ 0.52 (SiO_2_, dichloromethane/ethanol, 15:1, *v*/*v*). ^1^H-NMR (CDCl_3_; 600 MHz) δ_H_: 0.70 (m, 1H, H-5), 0.78 (s, 3H, CH_3_), 0.84 (s, 3H, CH_3_), 0.99 (s, 3H, CH_3_), 1.00 (s, 3H, CH_3_), 1.04 (s, 3H, CH_3_), 1.33 (m, 6H, 2× OCH_2_C*H*_3_), 1.06–2.01 (m, 24H, CH, CH_2_), 2.07 (d, *J* = 3Hz, 3H, H-30), 2.47 (m, 1H, H-19), 2.93 (s, 1H, C≡CH), 3.20 (m, 1H, H-3), 3.97 (d, *J* = 10.8 Hz, 1H, H-28), 4.05 (m, 4H, 2× OC*H*_2_CH_3_) 4.39 (d, *J* = 10.8 Hz, 1H, H-28), 5.43 (d, 1H, ^2^*J*_PH_ = 18 Hz, H-29). ^13^C-NMR (CDCl_3_; 150 MHz) δ_C_: 14.7; 15.4; 16.0; 16.1; 16.4; 18.2; 20.7; 25.4; 25.8; 26.9; 27.3; 28.0; 29.6; 34.1; 34.6; 37.1; 37.5; 38.6; 38.9; 40.8; 42.7; 46.6; 49.7; 50.2; 51.4; 55.3; 61.1; 61.2; 64.6; 74.6; 74.9; 78.9; 111.9 (d, ^1^*J*_CP_ = 183 Hz, C-29); 153.2; 166.8. ^31^P-NMR (CDCl_3_; 243 MHz) δ_P_: 18.25. IR (KBr, cm^−1^) ν_max_: 3360 (O-H), 2107 (C≡C), 1713 (C=O), 1230 (P=O), 961 (P-O-R), 753 (P-C). HR-MS (APCI, negative), *m*/*z* calcd. for C_37_H_58_O_6_P [(M − H)^−^] 629.3971, found 629.3974.

*29-Diethoxyphosphoryl-3*β*,28-dipropynoyloxy-lup-20E(29)-ene* (**6b**). Yield: 10%, m.p. 129–133 °C, R_f_ 0.74 (SiO_2,_ dichloromethane/ethanol, 15:1, *v*/*v*). ^1^H-NMR (CDCl_3_; 600 MHz) δ_H_: 0.81 (m, 1H, H-5), 0.87 (s, 3H, CH_3_), 0.90 (s, 3H, CH_3_), 0.91 (s, 3H, CH_3_), 0.99 (s, 3H, CH_3_), 1.04 (s, 3H, CH_3_), 1.33 (m, 6H, 2× OCH_2_CH_3_), 0.95–2.01 (m, 22H, CH, CH_2_), 2.08 (d, *J* = 3Hz, 3H, H-30), 2.47 (m, 1H, H-19), 2.88 (br. s, 1H, C≡CH), 2.93 (br. s, 1H, C≡CH), 3.97 (d, *J* = 10.8 Hz, 1H, H-28), 4,05 (m, 4H, 2× OCH_2_CH_3_) 4.39 (d, *J* = 10.8 Hz, 1H, H-28), 4.62 (m, 1H, H-3), 5.43 (d, 1H, ^2^*J*_PH_ = 18.6 Hz, H-29). ^13^C-NMR (CDCl_3_; 150 MHz) δ_C_: 14.7; 16,0; 16.1; 16.4; 18.1; 20.7; 23.5; 24.8; 25.5; 26.9; 27.9; 29.6; 33.7; 34.0; 34.6; 37.0; 37.4; 37.9; 38.3; 40.9; 42.7; 46.6; 49.4; 49.7; 50.1; 51.3; 55,3; 61.1; 61.2; 64.6; 74.1; 74.6; 74.9; 75.1; 83.6; 111.5 (d, ^1^*J*_CP_ = 186 Hz, C-29); 152.8; 153.2; 166.7. ^31^P-NMR (CDCl_3_; 243 MHz) δ_P_: 18.20. IR (KBr, cm^−1^) ν_max_: 3326 (C≡C-H), 2115 (C≡C), 1715 (C=O), 1232 (P=O), 963 (P-O-R), 754 (P-C). HR-MS (APCI, negative), *m*/*z* calcd. for C_40_H_58_O_7_P [(M − H)^−^] 681.3920, found 681.3908.

*29-Diethoxyphosphoryl-28-(2-butynoyloxy)-lup-20E(29)-en-3*β*-ol* (**7a**). Yield: 84%, m.p. 150–153 °C, R_f_ 0.46 (SiO_2,_ dichloromethane/ethanol, 15:1, *v*/*v*). ^1^H-NMR (CDCl_3_; 600 MHz) δ_H_: 0.69 (m, 1H, H-5), 0.78 (s, 3H, CH_3_), 0.84 (s, 3H, CH_3_), 0.99 (s, 6H, 2× CH_3_), 1.04 (s, 3H, CH_3_), 1.32 (m, 6H, 2× OCH_2_C*H*_3_), 0.8–2.10 (m, 24H, CH, CH_2_), 2.01 (s, 3H, C≡CCH_3_), 2.06 (d, *J* = 3 Hz, 3H, H-30), 2.47 (m, 1H, H-19), 3.20 (m, 1H, H-3), 3.94 (d, *J* = 10.8 Hz, 1H, H-28), 4.04 (m, 4H, 2× OC*H*_2_CH_3_), 4.33 (d, *J* = 10.8 Hz, 1H, H-28), 5.42 (d, 1H, ^2^*J*_PH_ = 18 Hz, H-29). ^13^C-NMR (CDCl_3_; 150 MHz) δ_C_: 3.9; 11.4; 14.7; 15.4; 16.0; 16.1; 16.4; 18.3; 20.7; 25.3; 25.8; 26.9; 27.3; 28.0; 29.1; 29.6; 34.1; 34.6; 37.1; 37.4; 38.6; 38.9; 40.9; 42.7; 46.5; 49.7; 50.2; 55.3; 61.1; 61.2; 64.0; 72.4; 78.9; 85.8; 111.0; 154.3; 167.0. ^31^P-NMR (CDCl_3_; 243 MHz) δ_P_: 18.33. IR (KBr, cm^−1^) ν_max_: 3414 (O-H), 2243 (C≡C), 1707 (C=O), 1247 (P=O), 1026 (P-O-C), 750 (P-C). HR-MS (APCI, negative), *m*/*z* calcd. for C_38_H_60_O_6_P [(M − H)^−^] 643.4127, found 643.4135.

*29-Diethoxyphosphoryl-3*β*,28-di(2-butynoyloxy)-lup-20E(29)-ene* (**7b**). Yield: 7%, m.p. 229–304 °C, R_f_ 0.71 (SiO_2_, dichloromethane/ethanol, 15:1, *v*/*v*). ^1^H-NMR (CDCl_3_; 600 MHz) δ_H_ (ppm): 0.79 (m, 1H, H-5), 0.87 (s, 3H, CH_3_), 0.89 (s, 6H, 2× CH_3_), 0.98 (s, 3H, CH_3_), 1.03 (s, 3H, CH_3_), 1.32 (m, 6H, 2× OCH_2_C*H*_3_), 0.8–2.10 (m, 23H, CH, CH_2_), 1.997 (s, 3H, C≡CCH_3_), 2.01 (s, 3H, C≡CCH_3_), 2.07 (d, *J* = 3 Hz, 3H, H-30), 2.47 (m, 1H, H-19), 3.93 (d, *J* = 11.1 Hz, 1H, H-28), 4.04 (m, 4H, 2× OC*H*_2_CH_3_), 4.33 (d, *J* = 11.1 Hz, 1H, H-28), 4.59 (m, 1H, H-3), 5.42 (d, 1H, ^2^*J*_PH_ = 18 Hz, H-29). ^13^C-NMR (CDCl_3_; 150 MHz) δ_C_: 3.9; 14.1; 14.7; 16.0; 16.1; 16.4; 16.5; 18.1; 20.7; 23.5; 25.5; 25.7; 26.9; 27.9; 29.6; 34.0; 34.6; 37.0; 37.4; 37.9; 38.3; 40.9; 42.7; 46.5; 49.7; 50.1; 50.2; 55.4; 61,1; 61.2; 64.0; 72.4; 72.8; 82.7; 85.0; 85.8; 111.4; 153.9; 154.3, 167.3. ^31^P-NMR (CDCl_3_; 243 MHz) δ_P_: 18.30. IR (KBr, cm^−1^) ν_max_: 2241 (C≡C), 1710 (C=O), 1242 (P=O), 1059 (P-O-C), 748 (P-C). HR-MS (APCI, negative), *m*/*z* calcd. for C_42_H_62_O_7_P [(M − H)^−^] 709.4233, found 709.4234.

*29-Diethoxyphosphoryl-28-cyclopropylpropynoyloxy-lup-20E(29)-en-3*β*-ol* (**8a**). Yield: 87%, m.p. 150–153 °C, R_f_ 0.50 (SiO_2,_ dichloromethane/ethanol, 15:1, *v*/*v*). ^1^H-NMR (CDCl_3_; 600 MHz) δ_H_: 0.69 (1H, m, H5), 0.70 (3H, s, CH_3_), 0.77 (3H, s, CH_3_), 0.98 (6H, s, 2× CH_3_), 1.02 (3H, s, CH_3_), 1.32 (6H, m, 2× OCH_2_C*H*_3_), 0.8–2.10 (29H, m, CH, CH_2_), 2.06 (3H, d, *J* =2.7 Hz, H30), 2.47 (1H, m, H19), 3.20 (1H, m, H3), 3.92 (1H, d, *J* = 10.8 Hz, H28), 4.04 (4H, m, 2 × OC*H*_2_CH_3_), 4.31 (1H, d, *J* = 10.8 Hz, H28), 5.42 (1H, d, ^2^*J*_HP_ =18 Hz, H29). ^13^C-NMR (CDCl_3_; 150 MHz) δ_C_: −0.1; 1.6; 9.8; 14.7; 15.3; 15.9 ; 16.5; 16.9; 18.8; 21.3; 23.2; 26.3; 27.5; 27.9; 28.5; 30.2; 32.1; 34.7; 35.2; 37.7; 38.0; 39.2; 39.4; 41.4; 43.2; 47.1; 50.3; 50.8; 55.8; 61.7; 64.4, 69.0; 79.5; 94.2; 112.5; 154.9; 167.9. ^31^P-NMR (CDCl_3_; 243 MHz) δ_P_: 18.35. IR (KBr, cm^−1^) ν_max_: 2243 (C≡C), 1707 (C=O), 1278 (P=O), 1038 (P-O-R), 750 (P-C). HR-MS (APCI, negative), *m/z* calcd. for C_40_H_62_O_6_P [(M − H)^−^] 669.4284, found 669.4270.

*29-Diethoxyphosphoryl-3*β*,28-dicyclopropylpropynoyloxy-lup-20E(29)-ene* (**8b**). Yield: 13%, m.p. 144–147 °C, R_f_ 0.76 (SiO_2_, dichloromethane/ethanol, 15:1, *v*/*v*). ^1^H-NMR (CDCl_3_; 600 MHz) δ_H_ (ppm): 0.79 (m, 1H, H-5), 0.85 (s, 3H, CH_3_), 0.88 (s, 6H, 2× CH_3_), 0.97 (s, 3H, CH_3_), 1.02 (s, 3H, CH_3_), 1.32 (m, 6H, 2× OCH_2_C*H*_3_), 0.8–2.10 (m, 33H, CH, CH_2_), 2.06 (d, *J* = 3 Hz, 3H, H-30), 2.46 (m, 1H, H-19), 3.91 (d, *J* = 11.1 Hz, 1H, H-28), 4.07 (m, 4H, 2× OC*H*_2_CH_3_), 4.31 (d, *J* = 10.8 Hz, 1H, H-28), 4.57 (m, 1H, H-3), 5.41 (d, 1H, ^2^*J*_PH_ = 18.6 Hz, H-30). ^13^C-NMR (CDCl_3_; 150 MHz) δ_C_: −1.6; −0.1; 8.1; 8.2; 8.5; 13.7; 15.0; 15.1; 15.3; 15.4; 15.5; 17.1; 19.7; 22.5; 24.7; 25.8; 25.9; 26.9; 28.6; 29.5; 30.6; 33.0; 33.6; 36.0; 36.4; 36.9; 37.3; 39.8; 41.6; 45.5; 48.7; 49.1; 54.4; 60.1; 60.1; 62.8; 67.4; 67.9; 81.6; 91.7; 92.6; 111.9; 152.9; 153.4; 166.0. ^31^P-NMR (CDCl_3_; 243 MHz) δ_P_: 18.33. IR (KBr, cm^−1^) ν_max_: 2223 (C≡C), 1710 (C=O), 1265 (P=O), 1014 (P-O-R), 799 (P-C). HR-MS (APCI, negative), *m/z* calcd. for C_46_H_66_O_7_P [(M − H)^−^] 761.4546, found 761.452.

### 3.5. Procedure for the Synthesis of Propagyloxycarbonyl Derivatives of Betulin ***9a*** and ***9b***

Compound **4** (0.29 g, 0.5 mmol) was dissolved in a mixture of 3 mL of dry benzene and 2.5 mL of pyridine and cooled to a temperature of about −5° C. To the mixture was added, dropwise, a solution of 1 mmol of propargyl chloroformate in 2.5 mL of benzene and then stirred for 18 h at room temperature. Then the volatile solvents were evaporated and the residue was dissolved in 3 mL of chloroform and washed with 10% sulfuric acid and water. Extracts were dried over anhydrous sodium sulfate, and then concentrated under reduced pressure. The crude product was purified by column chromatography (SiO_2_, dichloromethane/ethanol 15:1, *v*/*v*) to give the mono and disubstituted derivatives **9a** and **9b**.

*29-Diethoxyphosphoryl-28-propargyloxycarbonyloxy-lup-20E(29)-en-3*β*-ol* (**9a**). Yield: 63%, m.p. 140–142 °C, R_f_ 0.49 (SiO_2,_ dichloromethane/ethanol, 15:1, *v*/*v*). ^1^H-NMR (CDCl_3_; 600 MHz) δ_H_: 0.70 (m, 1H, H-5), 0.78 (s, 3H, CH_3_), 0.84 (s, 3H, CH_3_), 0.99 (s, 6H, 2× CH_3_), 1.05 (s, 3H, CH_3_), 1.33 (m, 6H, 2× OCH_2_C*H*_3_), 0.86–2.10 (m, 24H, CH, CH_2_), 2.08 (br. s, 3H, H-30), 2.47 (m, 1H, H-19), 2.56 (t, *J* = 2.4 Hz, 1H, C≡CH), 3.21 (m, 1H, H-3), 3.95 (d, *J* = 10.8 Hz, 1H, H-28), 4.05 (m, 4H, 2× OC*H*_2_CH_3_) 4.38 (d, *J* = 10.8 Hz, 1H, H-28), 4.76 (m, 2H, OCH_2_-C≡C), 5,43 (d, 1H, ^2^*J*_PH_ = 18 Hz, H-29). ^13^C-NMR (CDCl_3_; 150 MHz) δ_C_: 13.7; 14.3; 14.9; 15.0; 15.1; 15.4; 17.2; 19.7; 24.8; 25.9; 26.3; 27.0; 28.5; 28.6; 33.1; 33.5; 36.0; 36.1; 36.4; 37.6; 37.8; 39.8; 41.7; 45.7; 48.7; 49.2; 50.4; 54.2; 54.3; 60.1; 60.1; 66.0; 74.7; 77.9; 110.9; 154.0. ^31^P-NMR (CDCl_3_; 243 MHz) δ_P_: 18.30. IR (KBr, cm^-1^) ν_max_: 3437 (O-H), 2128 (C≡C), 1750 (C=O), 1250 (P=O), 969 (P-O-R), 789 (P-C). HR-MS (APCI, negative), *m/z* calcd. for C_38_H_60_O_7_P [(M − H)^−^] 659.4077, found 659, 4064.

*29-Diethoxyphosphoryl-3*β*,28-dipropargyloxycarbonyloxy-lup-20E(29)-en* (**9b**). Yield: 7%, m.p. 150–153 °C, R_f_ 0.70 (SiO_2_, dichloromethane/ethanol, 15:1, *v*/*v*). ^1^H-NMR (CDCl_3_; 600 MHz) δ_C_: 0.80 (m, 1H, H-5), 0.87 (s, 3H, CH_3_), 0.88 (s, 3H, CH_3_), 0.94 (s, 3H, CH_3_), 0.99 (s, 3H, CH_3_), 1.05 (s, 3H, CH_3_), 1.33 (m, 6H, 2× OCH_2_C*H*_3_), 0.82–2.10 (m, 23H, CH, CH_2_), 2.08 (br. s, 3H, H-30), 2,48 (m, 1H, H-19), 2.54 (br. s, 1H, C≡CH), 2.56 (br. s, 1H, C≡CH), 3.95 (d, *J* = 10.8 Hz, 1H, H-28), 4.06 (m, 4H, 2× OC*H*_2_CH_3_), 4.38 (m, 2H, H-3, H-28), 4.74 (m, 2H, OCH_2_-C≡C), 4.76 (m, 2H, OCH_2_-C≡C), 5.43 (d, 1H, ^2^*J*_PH_ = 18 Hz, H-29). ^13^C-NMR (CDCl_3_; 150 MHz) δ_C_: 13.7; 14.9; 15.1; 15.3; 15.4; 17.0; 19.7; 22.5; 24.7; 25.9; 26.8; 28.5; 28.7; 33.0; 33.5; 36.0; 36.4; 37.0; 37.3; 39.8; 41.7; 45.7; 48.7; 49.1; 54.0; 54.3; 60.1; 66.0; 74.4; 74.7; 76.4; 79.2; 85.1; 110.9; 153.5; 154.0. ^31^P-NMR (CDCl_3_; 243 MHz) δ_P_: 18.27. IR (KBr, cm^−1^) ν_max_: 2131 (C≡C), 1757 (C=O), 1263 (P=O), 1026 (P-O-R), 800 (P-C). HR-MS (APCI, negative), *m/z* calcd. for C_42_H_62_O_9_P [(M − H)^−^] 741.4131, found 741.4140.

### 3.6. Crystal Structure Determination

The X-ray diffraction data were collected on a Bruker D8 venture diffractometer equipped with an PHOTON100 CMOS detector using a Mo-K*α* radiation source (λ = 0.71073 Å) at 100 K under a continuous flow of cooled nitrogen gas. The structure was solved by direct methods using SHELXT [32] and refined by full-matrix least-squares fitting on *F*^2^ using MoPro program [33]. All non-hydrogen atoms were refined anisotropically. The hydrogen atoms were refined isotropically using X-H bond length restraints and X-H distance similarity restraints to favor their placement in the ideal position. The thermal motion parameters *U*_iso_ of hydrogen atoms were restrained to ride on 1.2 × *U*_eq_ for C-H and CH_2_ hydrogen atoms and 1.5 × *U*_eq_ for CH_3_ and OH hydrogen atoms. The O–H proton of compound **8a** was located from difference Fourier map. Detailed information regarding the crystallographic data and refinement is given in electronic supplementary information (ESI).

CCDC-1445930 contain the supplementary crystallographic date for this paper. These data can be obtained free of charge from the Cambridge Crystallographic Data Centre via www.ccdc.cam.ac.uk/data_request/cif.

### 3.7. Antiproliferative Assay in Vitro

#### 3.7.1. Cell culture

The synthesized compounds were tested against tumor cells T47D (human ductal carcinoma, mammary gland, derived from a metastatic site: pleural effusion, ATCC), SNB-19 (human glioblastoma, DSMZ-German Collection of Microorganisms and Cell Cultures, Braunschweig, Germany), and C32 (human melanoma; ATCC). The cultured cells were kept at 37 °C and 5% CO_2_. The cells were seeded (5 × 10^3^ cells/well/100 µL DMEM supplemented with FBS (Fetal Bovine Serum, Lonza) to a final concentration of 10% and streptomycin 10 mg/mL and penicillin 1000 IU/mL (Sigma, Saint Louis, MO, USA) using 96-well plates (Corning, New York, NY, USA).

#### 3.7.2. Biological Activity of Compounds

The antiproliferative effect of the synthesized compounds was determined using the Cell Proliferation Reagent WST-1 assay (Roche Diagnostics, Mannheim, Germany). The assay principle is based upon the reduction of the tetrazolium salt WST-1 to formazan by mitochondrial dehydrogenases in viable cells.

The tested compounds were exposed at various concentrations of each compound (0.1–100 µg/mL), and then cell viability was quantified by a cell proliferation assay. The generation of the dark yellow colored formazan was measured at 450 nm. The experiments were repeated in triplicate for each tested compound concentration. Calculations of the IC_50_ values were performed using GraphPad Prism 6 (GraphPad Software, San Diego, CA, USA). The cytotoxic effect of test compounds was compared with cisplatin (positive control).

## 4. Conclusions

The hydrolysis reaction of allylic phosphonate of betulin **3** in the presence of alkali metal hydroxide proceeds with migration of the double bond from the β,γ to the α,β-position, thus leading to afford vinylic phosphonates as a mixture of *E* and *Z* isomers (compounds **4** and **5**). The structure of the *Z* isomer was assigned based on the spectral analysis. The configuration *E* isomer (**4**) was confirmed by crystallographic analysis of the monocrystal and its cyclopropylpropynoic derivative (**8a**).

The introduction of a phosphonate group to the isopropenyl moiety of betulin **1** led to an increase of activity against the T47D and C32 cell lines. In relation to the human breast cancer (T47D) and glioblastoma (SNB-19), the derivatives containing this substituent at C29 exhibited higher activity than in the position C30. The evaluation of the in vitro cytotoxic activity of akynyl derivatives showed that, among the studied compounds, the 29-diethoxyphosphoryl-28-propynoyloxy-lup-20*E*(29)-en-3β-ol (**6a**) was the most active.

## Figures and Tables

**Table 1:** Chemical proportions *S*_x_ on the Hirshfeld surface around the molecule in the crystal structure and decomposition of intermolecular contacts in pairs of atom types *C*_xy_. The enrichment values *E*_xy_ are the ratios between *C*_xy_, the actual contact surfaces and those *R*_xy_ computed as if all contacts were randomly distributed [30]. *R*_xy_ equiprobable contacts are computed from *S*_x_ values using probability laws. The Hirshfeld surface analysis was carried out with MoProViewer software [31]. The hydroxyl hydrogen atom Ho was distinguished from the hydrophobic Hc atoms. The majors contact types and the most enriched are highlighted in bold characters.

Atom Type	Ho	C	O	Hc	P
% Surface	1.77	13.23	7.49	76.43	1.08
Ho	0.0				
C	0.1	1.0		contacts X···Y (%)	
O	1.8	1.7	0.0		
Hc	1.8	**21.9**	**11.3**	**58.2**	
P	0.0	0.0	0.0	2.2	0.0
Ho	0.00				
C	0.30	0.60		enrichment of contacts	
O	**6.54**	0.89	0.00		
Hc	0.62	1.11	1.00	0.99	
P	0.04	0.03	0.00	1.30	0.00

**Table 2:** Cytotoxic in vitro activity of betulin **1**, phosphonate derivatives **3**–**5**, **6a**–**9a**, **6b**–**9b**, and cisplatin against human cell lines: T47D, SNB-19, and C32.

Compound	Cytotoxic Activity IC_50_ (µg/mL)		
	T47D	SNB-19	C32
Betulin **1**	77.87 ± 4.71	23.52 ± 2.02	34.91 ± 5.37
**3**	58.00 ± 3.36	51.88 ± 0.76	2.15 ± 0.19
**4**	6.23 ± 0.53	5.92 ± 0.42	5.09 ± 0.1
**5**	0.89 ± 0.08	4.80 ± 0.09	5.25 ± 0.51
**6a**	0.44 ± 0.08	0.27 ± 0.09	0.38 ± 0.05
**6b**	2.84 ± 0.57	0.60 ± 0.06	2.71 ± 0.52
**7a**	9.63 ± 0.58	7.52 ± 0.28	4.96 ± 0.07
**7b**	13.92 ± 3.42	7.58 ± 1.00	4.9 ± 0.26
**8a**	8.18 ± 0.56	2.20 ± 0.78	4.69 ± 0.72
**8b**	79.52 ± 1.92	40.41 ± 6.85	9.44 ± 0.22
**9a**	49.38 ± 6.12	24.50 ± 12.62	5.95 ± 0.35
**9b**	Neg	0.49 ± 0.05	51.67 ± 4.76
cisplatin	55.95 ± 4.55	5.29 ± 0.12	4.29 ± 0.65

Neg—negative in the concentration used.

## Supplementary Materials

Supplementary materials can be accessed at: http://www.mdpi.com/1420-3049/21/9/1123/s1.
