# Supplementary material for: Betulin Phosphonates; Synthesis, Structure, and Cytotoxic Activity
Source: Molecules. 2016 Aug 26;21(9):1123. doi: 10.3390/molecules21091123 (PMC6273377; doi:10.3390/molecules21091123)
Supplement: Supplementary file 1 [file molecules-21-01123-s001.pdf]

Supplementary Materials: Betulin Phosphonates; Synthesis, Structure, and Cytotoxic Activity

Elwira Chrobak, Ewa Bębenek, Monika Kadela-Tomanek, Małgorzata Latocha, Christian Jelsch, Emmanuel Wenger and Stanisław Boryczka

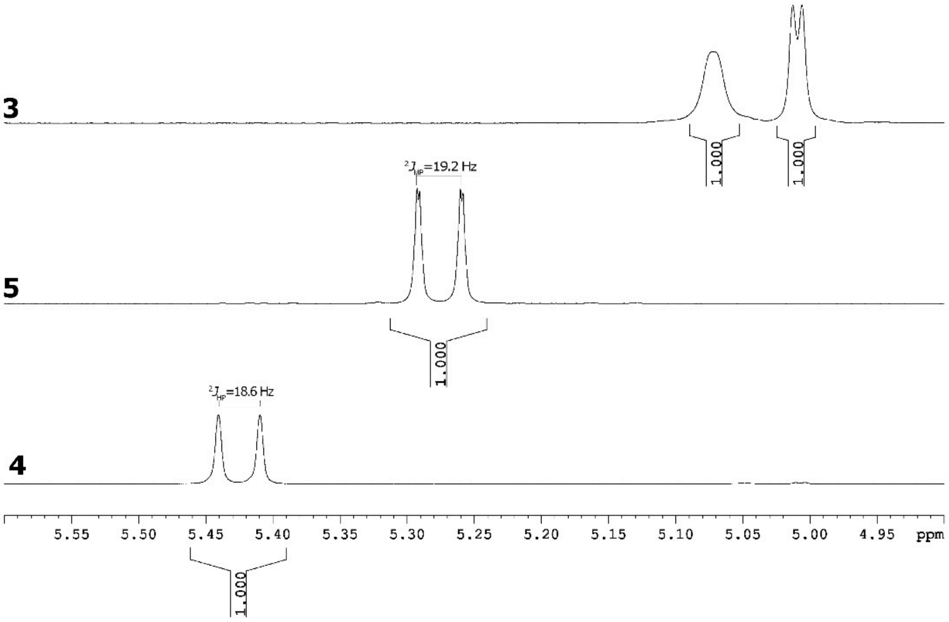

Figure S1. Expanded ^{1}H-NMR; the signals of proton H30 (compound **3**) and H29 (compound **4** and **5**); comparison of the chemical shift and integration.

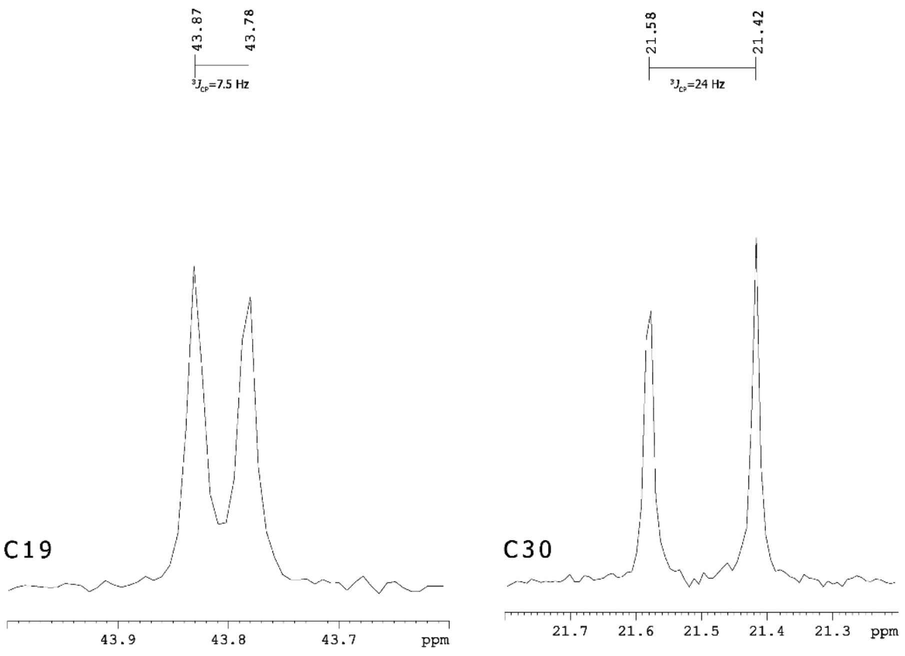

Figure S2. Expanded ^{13}C-NMR; the signals of carbon C19 and C30 of isomer **5**.

Crystal Structure Description

Single crystals of the compound **8a**, suitable for the X-ray diffraction studies, were grown up from a saturated tetrahydrofuran (THF) solution.

The compound 29-diethoxyphosphoryl-28-cyclopropylpropynoyloxy-lup-20*E*(29)-en-3β-ol **8a**, crystallizes in orthorhombic space group $P2_12_12_1$ (a = 7.5250(12), b = 21.028(4) and c = 24.187(4) Å). Crystal parameters, data collection and refinement details, are collected in Table S1. As it is shown on Figure S3, the unit cell contains four molecules of compound **8a** (Z = 4).

The *a* unit cell axis length is significantly shorter than the two others and symmetric molecules related by translation +**a** and –**a** interact with the molecule on each side (Figure S4).

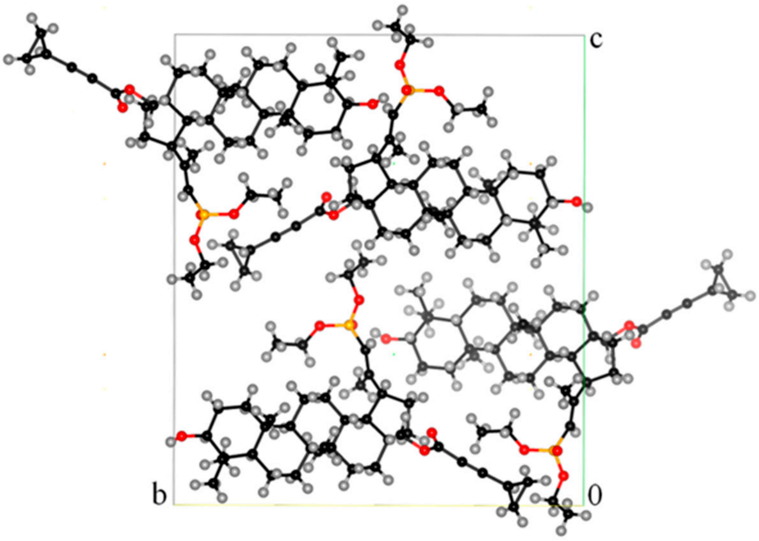

Figure S3. View along the ***a*** axis of the four molecules in the unit cell.

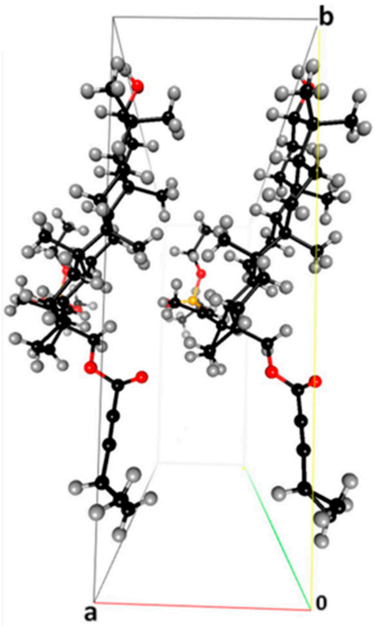

Figure S4. Auto-stereogram (crossed-eyes) of **a** dimer of molecules viewed along the **c** axis. The molecules are related by unit cell vector **a** translation [*a* = 7.5250(12)].

Table S1. Crystal data and structure refinement details for compound **8a**: 29-diethoxyphosphoryl-28-cyclopropylpropynoyloxy-lup-20*E*(29)-en-3β-ol.

Formula	$C_{40}H_{62}O_6P$
formula wt	667.85
temperature [K]	100(2)
wavelength [Å]	0.71073
crystal system	orthorhombic
space group	P212121
a [Å]	7.5250(12)
b [Å]	21.028(4)
c [Å]	24.187(4)
volume $(Å)^3$	3827.1(11)
Z	4
density (calcd) [g/cm^3]	1.159
absorption coeff (mm^{-1})	0.115
F(000)	1452
crystal size [mm^3]	0.20 × 0.23 × 0.25
θ range [deg]	2.11 to 27.42
reflection collected	48 091
data (*R*int)	8 693 (0.0596)
completeness , d_{min} [%, Å]	99.7, 0.77
weighting scheme	1/(8.5 σ_I^2)
restraints/parameters	283/676
GoF on F^2	1.00
$R1(F)$ $[I > 2\sigma(I)]$/all data	0.53/0.60
$wR2(I)$ $[I > 2\sigma(I)]$/all data	0.104/0.107
Largest peak and hole (e/Å^3)	0.84, −0.54

Table S2. List of strong O-H···O and weak C-H···O hydrogen bonds in the crystal structure.

D	H	A	DH	HA	DA	DHA	Symmetry
O1	HO1	O4	0.8203	1.914	2.7211(4)	167.9	3_546
C23	H23c	O1	0.9605	2.631	2.9798(5)	102.0	1_555
C22	H22b	O2	0.9695	2.462	2.8475(5)	103.4	1_555
C30	H30a	O4	0.9603	2.427	3.1962(5)	136.9	1_555
C37	H37b	O6	0.9702	2.568	3.0200(5)	109.0	1_555
C36	H36A	O6	0.972	2.652	3.5891(6)	162.1	3_656
C29	H29	O1	0.9301	2.494	3.4000(4)	164.8	3_656
C22	H22a	O3	0.9701	2.599	3.1762(4)	118.3	1_455

D: donor, A: acceptor. Distances DH, DA, Ha are in Å and DHA angles are in degrees. Symmetry: 1: 'x, y, z', 2: '−x + ½, −y, z + ½', 3: '−x, y + ½, −z + ½', 4: 'x + ½, −y + ½, −z'.

^{1}H- and ^{13}C-NMR Spectra for Selected Compounds

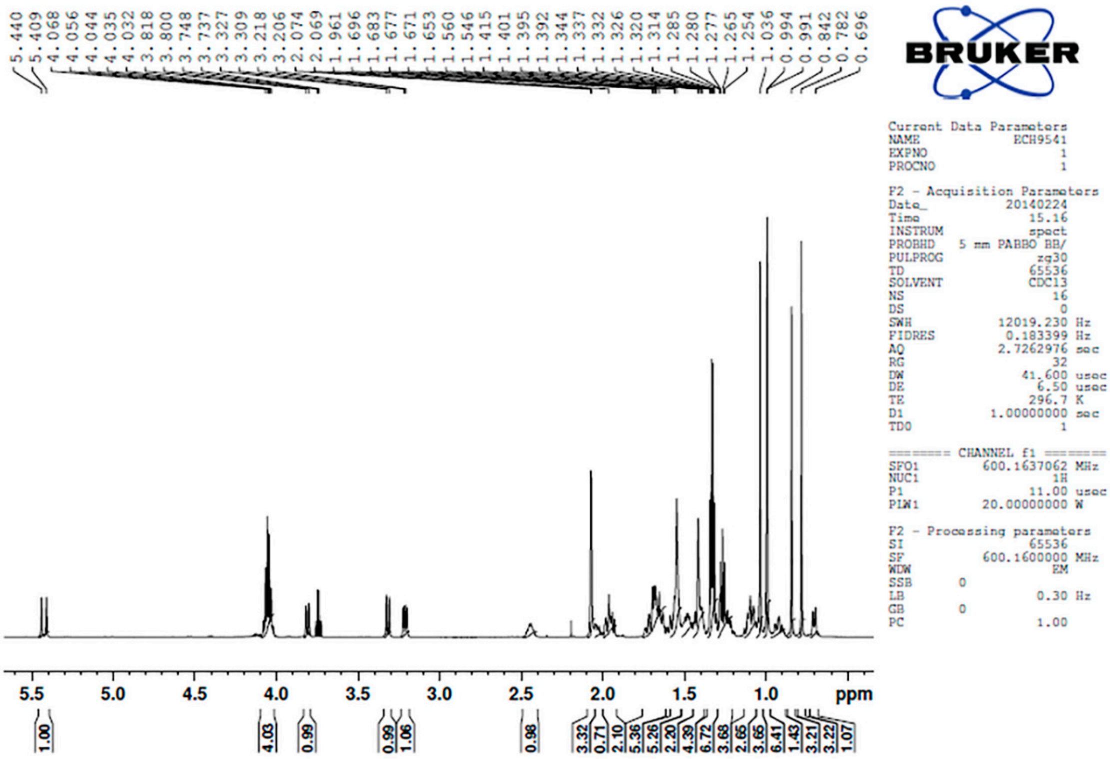

Figure S5. Compound **4**—^{1}H-NMR.

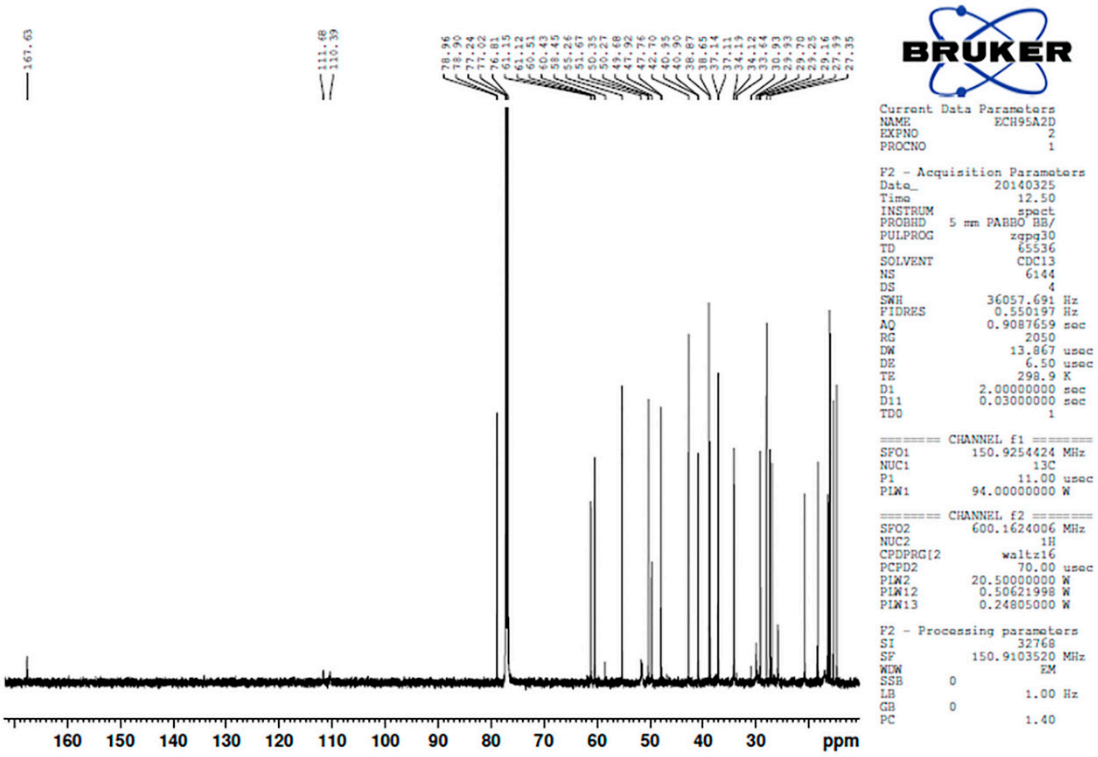

Figure S6. Compound **4**—^{13}C-NMR.

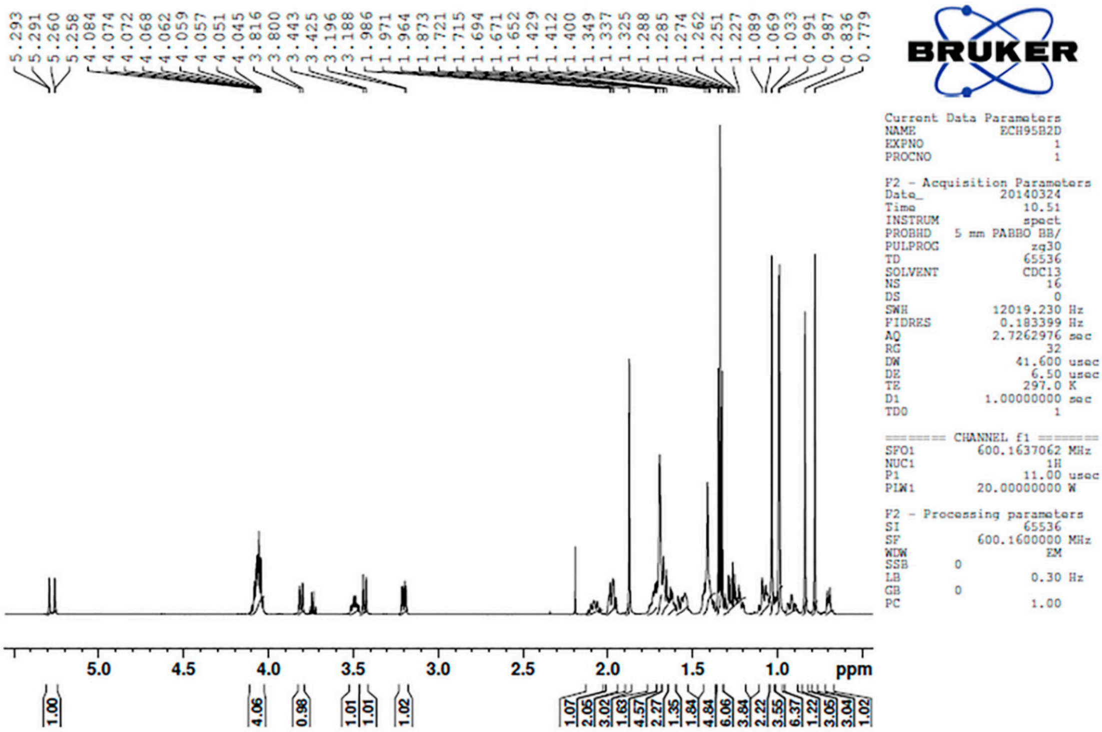

Figure S7. Compound **5**—^{1}H-NMR.

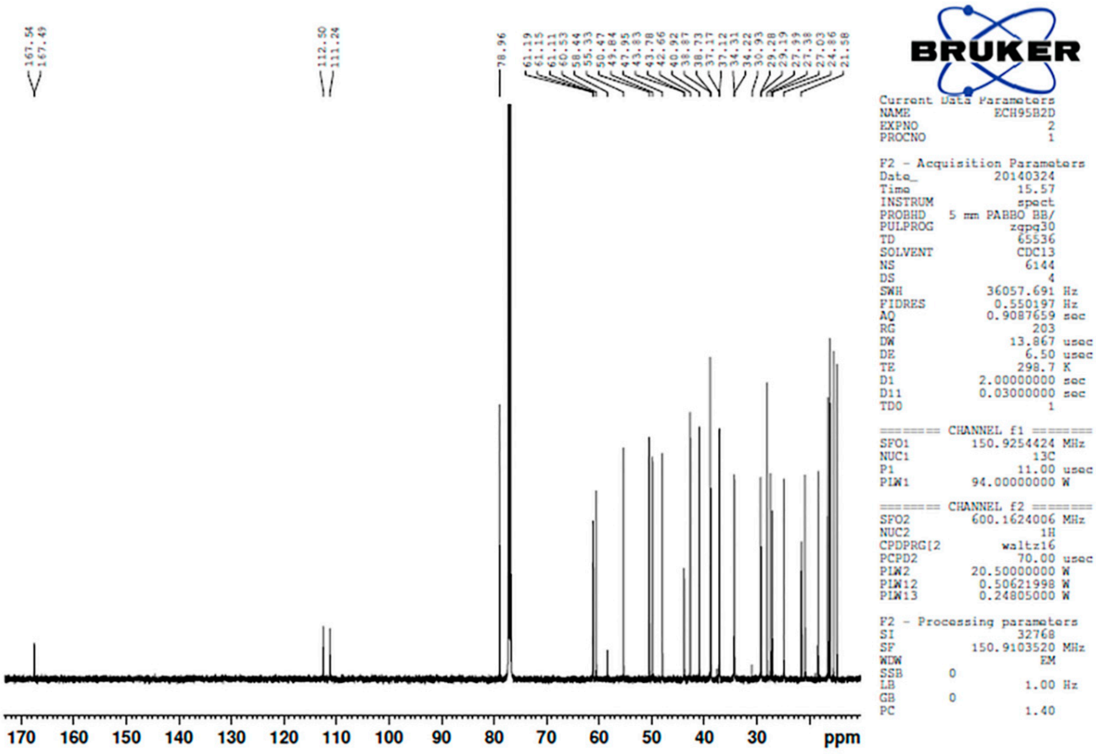

Figure S8. Compound **5**—^{13}C-NMR.

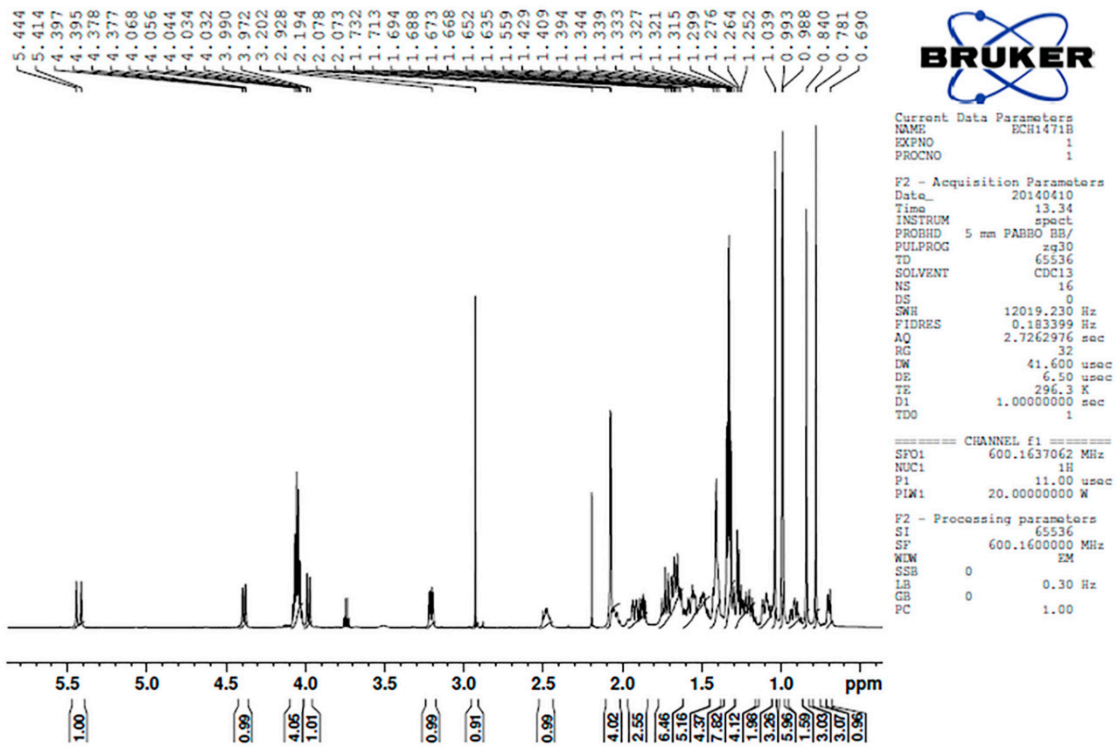

Figure S9. Compound **6a**—^{1}H-NMR.

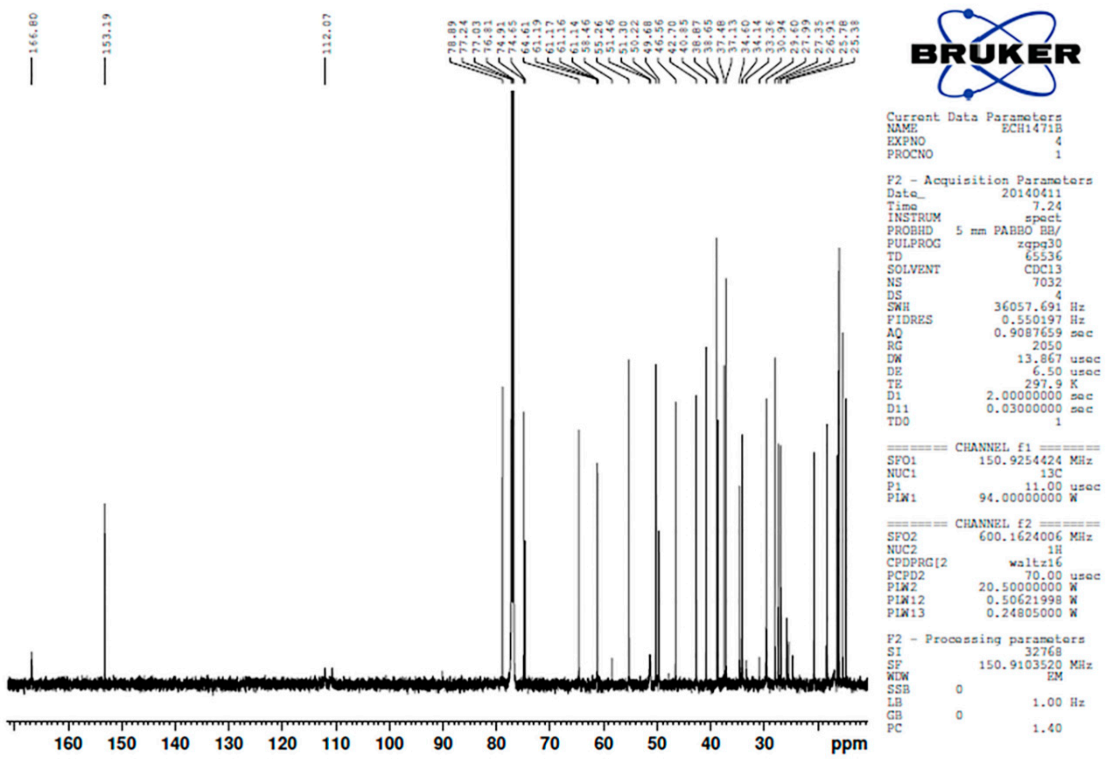

Figure S10. Compound **6a**—^{13}C-NMR.

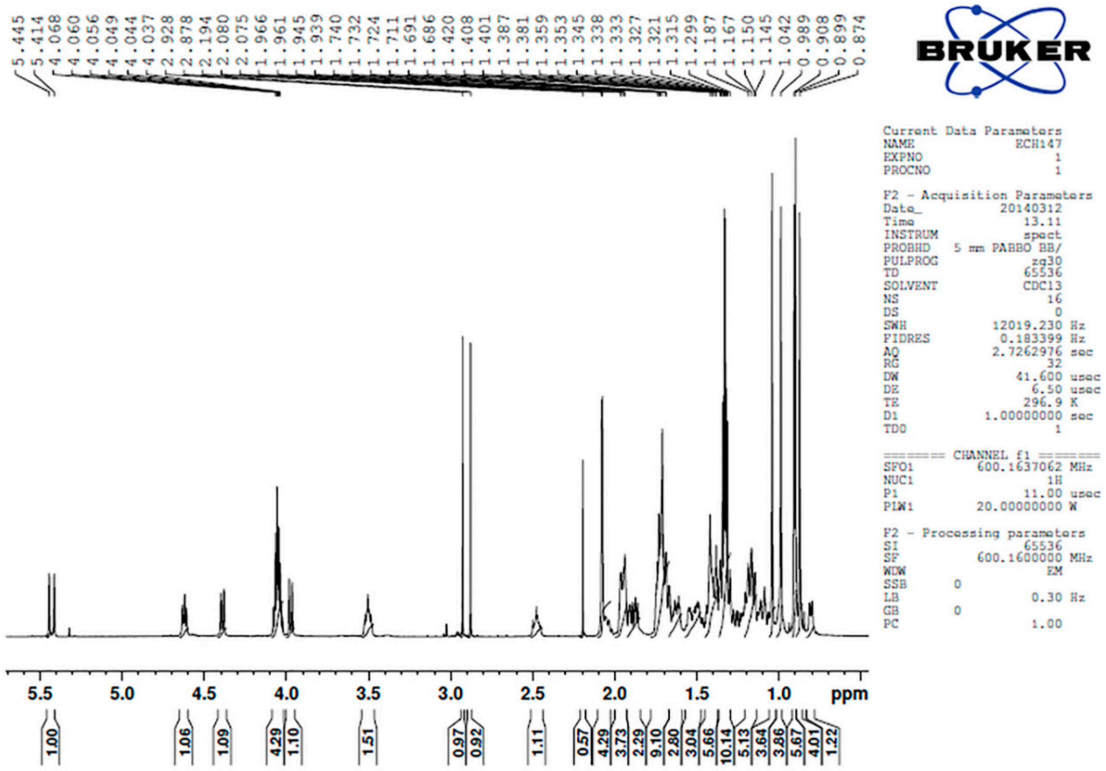

Figure S11. Compound **6b**—^{1}H-NMR.

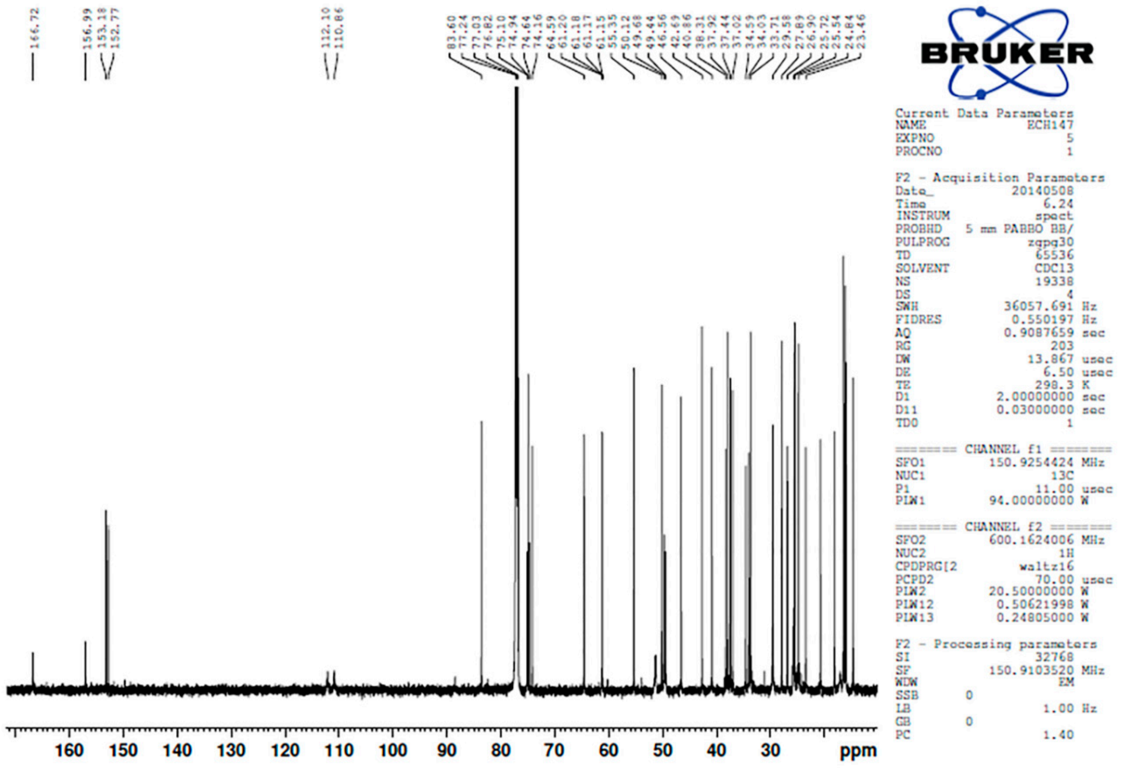

Figure S12. Compound **6b**—^{13}C-NMR.
